# Consequences, Motives, and Expectancies of Consumption as Predictors of Binge Drinking in University Women

**DOI:** 10.3389/fpsyg.2022.862334

**Published:** 2022-04-05

**Authors:** María-Teresa Cortés-Tomás, José-Antonio Giménez-Costa, Patricia Motos-Sellés, María-Dolores Sancerni-Beitia

**Affiliations:** ^1^Department of Basic Psychology, Faculty of Psychology, University of Valencia, Valencia, Spain; ^2^Department of Methodology of the Behavioral Sciences, Faculty of Psychology, University of Valencia, Valencia, Spain

**Keywords:** risky consumption, binge drinking, motives, expectancies, consequences, university women

## Abstract

The increasing presence of women, especially university women, in risky alcohol consumption such as Binge Drinking (BD), which is associated with gender-specific biopsychosocial problems, makes it necessary to analyze the variables underlying BD in order to adjust possible interventions more in line with their reality. The motives and expectancies of this pattern of consumption, as well as the consequences derived from it, are some of the variables that are shown to have the greatest weight in the prediction of BD. In the present study we analyze, on the one hand, the performance of these variables among college women with alcohol use, and on the other hand, which of these variables allow us to classify BD. A total of 501 female university consumers of alcohol (mean age 19.02 years) were assessed. Specifically, they completed a self-report of alcohol consumption (77.1% engage in BD), the Expectancy Questionnaire (EQ), the Drinking Motives Questionnaire (DMQ-R) and the Alcohol Consumption Consequences Evaluation (ACCE). BD female students scored significantly higher on these instruments, except for compliance motives. The logistic regression analysis carried out to estimate the probability of performing BD using the social and conformity motives, the ACCE and positive expectancies correctly estimated (χ^2^_8_ = 9.149, *p* < 0.33) 88.6% of the cases and explained 26.2% of the BD. Thus, young women with a level of consequences classified as high risk (>25 in ACCE) have a 3.55-fold increase in the probability of having BD, compared to women classified as low risk by the ACCE. On the other hand, women classified as moderate risk by the ACCE have a 4.77-fold increase in the probability of having BD. In the case of social motives and positive expectancies, their increase multiplies by 1.165 and 1.024, respectively, the probability of having BD. The results of this study highlight the importance of adapting preventive measures to the consequences experienced by BD university students, especially in relation to the social motives and positive expectancies that modulate decision-making when engaging in this pattern of consumption.

## Introduction

Binge drinking (BD) constitutes one of the most frequent patterns of risky consumption among young people (National Institute on Alcohol Abuse and Alcoholism [NIAAA], 2004; Observatorio Español de las Drogas y las Adicciones, 2021), with a special prevalence among university students (Grant et al., 2015; Johnston et al., 2015; Amare and Getinet, 2019; Crawford et al., 2019; Haardörfer et al., 2021).

Even though men as a whole continue to outnumber women in terms of the number of BD consumers and the amount of alcohol consumed (White et al., 2015), the number of women engaging in this pattern of consumption in the recent years is increasing and sometimes even surpassing that of men (White et al., 2015; Grant et al., 2017; Iwamoto et al., 2018; Wilsnack et al., 2018; Kang et al., 2020; Alves et al., 2021; Aston et al., 2021; Observatorio Español de las Drogas y las Adicciones, 2021). In the case of adolescent girls aged 18 in Spain who consume alcohol, 61.6% admit to having been intoxicated in the past year, compared to 59.2% of partners of the same age (Observatorio Español de las Drogas y las Adicciones (OEDA), and Delegación del Gobierno para el Plan Nacional sobre Drogas, 2020). Moreover, in college women from different Spanish universities (Cortés et al., 2017) the percentage of them who engage in BD reaches 60.4%, compared to 39.6% of their male counterparts.

This new reality places women at a higher risk of suffering harmful consequences due to their physiological characteristics (weight, body composition, etc.) and their body’s rate of absorption and metabolism of alcohol (Ashley et al., 1977; Erol and Karpyak, 2015). Several effects of alcohol have been documented in university women (Patrick et al., 2020), such as menstrual cycle alterations and infertility (Van Heertum and Rossi, 2017), memory loss (Baildon et al., 2021), feelings of sadness and depression (Barnett et al., 2014; Patrick et al., 2020), increased risk of sexually transmitted infections (Hutton et al., 2008), violence from their partners (Stanesby et al., 2018), as well as increased susceptibility to infectious, cardiac, and liver diseases (Hashimoto and Wiren, 2008; Kirpich et al., 2017).

This greater presence of women who engage in BD in combination with the consequences derived from this pattern of consumption justify the need to conduct more detailed analyses of the factors that predict this risky consumption (LaBrie et al., 2009; Brady et al., 2016; Iwamoto et al., 2018).

On the one hand, social learning theories suggest that cognitions -motives and expectancies- influence behavior (Bandura and McClelland, 1977; Maisto et al., 1999) and on the other hand, research with university students has shown that the consequences experienced when consuming alcohol affect the way in which young people evaluate the experiences of their consumption, as well as the subsequent consumption behavior, so it becomes another source of cognition to consider (Mallett et al., 2008; Patrick and Maggs, 2011; Yurasek et al., 2011; Merrill et al., 2013; Barnett et al., 2015).

Research regarding the role of consumption expectancies as a determining factor in university women’s BD behavior (Lyons and Willott, 2008; Watts et al., 2015; Jacobs and Jacobs, 2016; Kim, 2018) concludes that positive expectancies related to social aspects are strong predictors of BD. Lyons and Willott (2008), Young et al. (2015), and Iwamoto et al. (2016) allude to the fact that consuming alcohol produces feelings of power, a trait that they believe will help them fit in with their peer group and gain social attention. Furthermore, Iwamoto et al. (2018) show that positive expectancies related to improving sexual experiences and reducing tension are related to BD.

Findings regarding negative expectancies have been less consistent, given that an inversely proportional relationship can be seen between these and alcohol consumption, regardless of gender (Nicolai et al., 2010; Ramirez et al., 2020), although when risky consumption is evaluated, this relationship becomes positive (Zamboanga et al., 2010; Pabst et al., 2014; Patrick et al., 2016; Alves et al., 2021). Different authors (Zamboanga and Ham, 2008; Bacio, 2021) explain this positive association between negative expectancies and risky consumption, such as BD, by indicating that university students might find the effects that researchers label as negative to be attractive.

The motives behind alcohol consumption are considered to be closer to carrying out the activity rather than to expectancies, which tend to have a more distant influence on it (Cooper, 1994). In this regard, it is possible for a person to expect alcohol consumption to relieve stress, but to not consume it when he/she feels stressed. However, a person is much more likely to be motivated to consume alcohol after experiencing how drinking alcohol helped them cope with stress. As Capron and Schmidt (2012) conclude, motives mainly feed on the real experiences (consequences) that people experience with alcohol consumption, and not only on the beliefs or expectations about what will happen after its use.

To date, several studies with general university population (both males and females) report that coping motives stand out for their greater weight in explaining both BD (Herschl et al., 2012; Patrick et al., 2017; Terry-McElrath et al., 2017), as well as problems derived from this consumption behavior (Cooper et al., 2016; Bacio, 2021; Bresin and Mekawi, 2021; Richards et al., 2021). Enhancement and social motives also share an important weight in the prediction of BD (Patrick et al., 2021; Richards et al., 2021). However, when dealing with larger consumption amounts both tend to lose explanatory weight, especially social motives (Tragesser et al., 2007; Cooper et al., 2008; Kuntsche and Cooper, 2010), even though people who mainly drink for social motives are more vulnerable to social norms regarding alcohol consumption. For this reason, they show a higher risk of developing excessive consumption patterns and experiencing associated problems when they are integrated into a subculture of heavy alcohol consumption, such as university (Watts et al., 2015; Jacobs and Jacobs, 2016; Kim, 2018; Bainter and Ackerman, 2021).

On the other hand, when reviewing the research related to conformity motives, there are some inconsistencies in university population (Cooper et al., 2016; Bresin and Mekawi, 2021). Several studies have observed a negative and weak relationship of this motives with alcohol consumption (Grant V. V. et al., 2009; Richards et al., 2021), and others have found it to have a positive and weak relationship with problems derived from consumption (Vernig and Orsillo, 2015; Wahesh and Lewis, 2015; Bacio, 2021; Richards et al., 2021).

Studies carried out exclusively with women at university show similar results, highlighting coping motives for their greater predictive weight, both in terms of patterns of risky alcohol consumption, and of the consequences derived from them (LaBrie et al., 2007; O’Brien et al., 2008; Kenney et al., 2015; Hussman, 2018; Kim, 2018). It is followed by enhancement motives (LaBrie et al., 2007; O’Brien et al., 2008; Loxton et al., 2015), and lastly, social motives (LaBrie et al., 2007; O’Brien et al., 2008). Only the work of Hussman (2018), in which she includes specific norms related to the female gender, shows that conformity motives obtain a relevant weight when explaining BD behavior and its consequences.

Until now, research has focused mainly on what young people hope to achieve with alcohol consumption -outcome expectancies-, on the reasons they have for drinking -consumption motives- and on the positive association of both aspects with BD and its consequences (Neighbors et al., 2003; Read et al., 2004). However, there is still a lack of research in university students in terms of the evaluation of the influence of consequences on BD (Lee et al., 2011). Corbin et al. (2008) have shown that the consequences of alcohol use represent a unique variation in predicting this drinking pattern after controlling for drinking expectancies, suggesting that consequences may differ from expectations and justify a separate evaluation (Lee et al., 2011). Likewise, Capron and Schmidt (2012) conclude that consumption consequences regarded as positive by university students predict a higher percentage of BD variance above the social and enhancement motives for consumption. Longitudinal studies have also observed that experiencing positive consequences derived from consumption predicts the likelihood of university students engaging in BD (Patrick and Maggs, 2011; Park et al., 2013; Patrick et al., 2016).

In this study, after evaluating the type of consequences, motives, and expectancies that define BD in female university students, we analyzed which of these variables help classify women in this consumption pattern. All this will allow specifying the best type of intervention that can be applied in this group. To develop this general objective, the variables considered for carrying out said classification will be described first, then the contrast will be continued based on the manifestation of the BD/non-BD behavior, and finally, an estimation will be made of the probability of occurrence of the behavior from these variables.

## Materials and Methods

### Participants

The sample of this study consists of 501 alcohol-consuming female university students between 18 and 20 years old –18 years old (25.9%, *n* = 130), 19 years old (33.5%, *n* = 168), and 20 years old (40.5%, *n* = 203) with a mean age of 19.15 years (SD = 0.80). The age of onset for alcohol consumption is 15 (SD = 1.46) and 87.4% (*n* = 438) have engaged in BD and 12.6% (*n* = 63) no BD. The entire sample consisted of 898 participants, however, 397 cases were excluded because missing values and only the sample for which complete information was available was used.

Participants were recruited using the “snowball” method. The researchers visited classes of three Degrees of the University of Valencia with the highest female ratio–(Psychology, Language Therapy and Social Work). In all cases, they asked for student’s voluntary collaboration. Students who agreed to participate were summoned another day to fill out the questionnaire. Prior to the completion of the tests, all young people signed an informed consent, where the research objectives were clearly reflected, and the anonymity of the data was guaranteed. The instrument was filled out in the presence of one of the interviewers.

### Variables

Sociodemographic: Sex, chronological age, and age of onset of alcohol consumption have been included.

Pattern of Consumption/BD: A self-report form of the last 6 months was used. This time interval makes it possible to account for the intermittent consumption (with periods of non-consumption that can exceed 30 days) carried out by young people (Townshend and Duka, 2005; Courtney and Polich, 2009).

This self-report form is an adaptation of the Timeline Followback (TLFB) by Sobell and Sobell (1996). For each day, the start and end time of each episode of consumption is recorded as well as the number of consumed SDUs (Standard Drinking Units). To help calculate the SDUs, participants were presented with a figure containing the equivalences between alcoholic beverages, their volume, and the number of respective SDUs. From the information offered by the participants in this self-report, the following variables were generated:

Maximum SDUs consumed: the consumption episode with the highest amount of SDUs ingested was selected.

Engagement or not in BD: the participants were classified as BD or non-BD based on the SDUs consumed in the episode of maximum consumption and the number of hours in which the consumption took place. The proposal of the National Institute on Alcohol Abuse and Alcoholism [NIAAA] (2004) was used as criterion to define the BD in this study, but in this case the grams of alcohol proposed by the original definition were adjusted to the Spanish SDU (1 SDU = 10 grams). Thus, women who consumed six or more SDUs in an interval of 2–3 h were classified as BD.

Expectancy Questionnaire (EQ, Leigh and Stacy, 1993; Spanish adaptation from Camacho et al., 2010). The scale consists of 34 items in a 6-point Likert format (0 = Never a 5 = Always) measuring positive and negative expectancies about alcohol consumption. Items take the form of short phrases prefaced by When I drink alcohol… Respondents were instructed to indicate the likelihood that the indicated effects or consequences would happen to them when they drink. The eight scales included in the questionnaire (grouped into two factors) are listed in Table 1 together with their reliability coefficients for the present sample. The original questionnaire presented adequate reliability coefficient, ranging from 0.73 for the tension-reduction scale to 0.91 for the sex scale; as well as 0.94 for positive expectancies and 0.88 for negative expectancies. The adaptation to Spanish obtained similar results, both in the first order factors (0.75–0.93) and in the second order factors (0.95 for positive expectancies and 0.91 for negative expectancies).

**TABLE 1:** Reliability coefficients of the evaluated sample.

	Second-order factors	First-order factors	Alpha coefficient
Expectancy questionnaire (EQ)	Positive expectancies 0.93	Positive social	0.89
		Fun	0.89
		Sex	0.89
		Tension reduction	0.78
	Negative expectancies 0.87	Negative social	0.73
		Emotions	0.80
		Physical effects	0.77
		Cognitive effects	0.76
Drinking motives questionnaire–revised (DMQ-R)		Social	0.74
		Enhancement	0.86
		Conformity	0.87
		Coping with anxiety	0.65
		Coping with depression	0.91
ACCE			0.93

Drinking Motives Questionnaire–Revised (M-DMQ-R) (Grant et al., 2007; Spanish adaptation from Mezquita et al., 2011). It consists of 28 items, each contributing to one of five subscales. Using a five-point scale (from 1 -almost never/never- to 5 -almost always/always-) participants were asked to decide how frequently their own drinking is motivated by each of the reasons listed. Mezquita et al. (2011) obtained internal consistency values between α = 0.88 of coping with depression and α = 0.63 of coping with anxiety. Table 1 shows the reliability coefficients obtained in this sample.

Alcohol Consumption Consequences Evaluation–(ACCE) (Sancerni-Beitia et al., 2020). It is a one-dimensional questionnaire with 43 dichotomous items (Yes/No) that comprise a wide range of consequences derived from their alcohol consumption during the last year, ordered according to two parameters: severity (identifies which consequences warn of problems of special relevance to be considered in terms of prevention/intervention) and discrimination (indicating that small differences in the trait are associated with a large difference in the probability of accepting the item). In its development it showed adequate psychometric properties: α = 0.93. This instrument allows individuals to be classified into three risk groups (low, moderate, and high risk): low risk (coded with 1) when scores are under 20 points, moderate (coded with 2) between 21 and 24 points, and high (coded 3) when scores are over 25 points.

### Procedure

For data collection, eight people received training in administering the instrument, so correct completion of it was guaranteed. All of them had two guided practices under the tutelage of the signatories of this study.

The study was conducted in compliance with Spanish legislation (Organic Law 3/2018, of December 5) and the code of ethics for research involving human subjects, as outlined by the University of Valencia Human Research Ethics Committee. The survey used in this study is completely anonymous. In addition, the survey itself includes an introduction that specifies the objectives to be achieved and the benefits it can bring, as well as an explicit reference to compliance with the current Data Protection Law. The last part of the introduction includes a paragraph in which the person indicates that they agree to participate voluntarily in the study.

### Data Analysis

Using the statistical package IBM SPSS Statistics 26, descriptive analyses of the following variables were carried out according to the first specific objective: age of onset of alcohol consumption, expectancies, consumption motives, and consequences experienced after consumption. *T*-tests were performed to contrast the means of the mentioned variables in the groups of young people who did or did not engage in BD according the second specific objective.

Next, a binary logistic regression was conducted to obtain an estimate of the probability of BD from three independent quantitative variables -expectancies, ACCE, and motives- according to the third objective. With this regression it was possible to estimate the probability of performing BD based on the set of independent variables, quantifying the importance of the relationship, and classifying the young women into the different established categories.

As stated in the introduction, expectancies, motives, and consequences can influence BD, to a greater or lesser extent. In this study, the relationships of these variables with the dependent variable BD is analyzed in combination, with the value 1 being assigned to the people of the group of interest, that is, to the group that consumes intensively (consume 60 grams or more) and 0 to the rest.

Given that ACCE classifies risk into three groups, two dummy variables were constructed. The reference category in both cases was the group of low-risk women, the ACCE1 dummy variable included high-risk young women, and the ACCE2 dummy variable those of moderate risk.

## Results

Table 2 shows the means and standard deviations of expectancies, motives, and consequences for the full sample and differentiating between BD and non-BD, as well as the differences between their means.

**TABLE 2:** Mean scores on each bing drinking subscale and between-group comparison.

		Full sample mean (SD) *n* = 501	Non-BD mean (SD) *n* = 63	BD mean (SD) *n* = 438	t	d
ACCE		21.45 (10.23)	11.25 (9.12)	22.56 (9.50)	8.87**	1.19
Expectancies	Positive	47.90 (20.21)	30.87 (18.22)	47.18 (18.93)	6.42**	0.86
	Negative	20.39 (12.63)	15.03 (13.45)	19.88 (12.01)	2.95**	0.39
Motives	Social	14.00 (4.26)	11.16 (3.72)	14.40 (4.20)	5.81**	0.78
	Enhancement	11.48 (4.90)	8.65 (3.94)	11.86 (4.89)	5.84**	0.67
	Conformity	6.27 (2.74)	6.48 (3.39)	6.24 (2.64)	0.64	0.09
	Coping-with-anxiety	5.80 (2.44)	5.05 (2.05)	5.89 (2.45)	2.95**	0.35
	Coping-with-depression	13.00 (5.92)	11.00 (3.63)	13.25 (6.08)	4.01**	0.38

**p < 0.01.

The logistic regression analysis includes all the independent variables: ACCE, positive and negative expectancies, and the five groups of motives -social, enhancement, conformity, coping with anxiety, and coping with depression. This model makes it possible to make a correct estimate (χ^2^_8_ = 8.33, *p* < 0.402) of 88.8% of the cases and accounts 27.3% of the BD variable (R Nagelkerke), although not all the independent variables are useful. Specifically, enhancement (Wald = 0.084, *p* = 0.772), coping with anxiety (Wald = 0.017, *p* = 0.895), and depression motives (Wald = 0.132, *p* = 0.717), as well as negative expectancies (Wald = 3.748, *p* = 0.053) were not significant. By including only social and conformity motives, ACCE, and positive expectancies as predictors, a model is obtained that allows a correct estimate to be made (χ^2^_8_ = 9.149, *p* < 0.33) of 88.6% of the cases and an explained variance percentage of 26.2%, Table 3.

**TABLE 3:** Classification table.

	Predicted bring drinking			
		No	Yes	Correct percentage
Observed	No	8	55	12.7
Binge drinking	Yes	2	437	99.5
Global percentage				88.6

Logistic regression revealed that the ACCE variables, positive expectancies and social and conformity motives had a statistically significant effect on the probability of developing risky drinking behavior. Table 4 summarizes the results of the logistic regression.

**TABLE 4:** Logistics regression on binge drinking (BD) according to ACCE, positive expectancies, and social and conformity motives.

							95% C.I. for Exp (B)	
	B	S.E.	Wald	df	p	Exp (B)	Lower	Higher
ACCE			11.917	2	0.003			
ACCE (1)	1.267	0.470	7.255	1	0.007	3.551	1.412	8.927
ACCE (2)	1.563	0.623	6.304	1	0.012	4.773	1.409	16.172
Social motives	0.152	0.048	10.082	1	0.001	1.165	1.060	1.127
Conformity motives	−0.177	0.054	10.928	1	0.001	0.838	0.754	0.930
Positive expectancies	0.023	0.010	6.059	1	0.014	1.024	1.005	1.043
Constant	−0.262	0.505	0.270	1	0.603	0.769		

The probability of engaging in heavy alcohol consumption is 3.55 times higher in the high-risk group (ACCE1) than in the low-risk group. In the case of the moderate risk group (ACCE2) this probability is 4.77 times higher.

On the other hand, an increase in the score for social motives multiplies the probability of engaging in BD by 1.165, and an increase in the score for positive expectancies multiplies this probability by 1.024. However, conformity motives seem to work inversely, given that low scores on this variable increase (OR = 0.838) the probability of BD.

## Discussion

The aim of this study was not only to gain a greater knowledge of the cognitive factors that underlie the pattern of alcohol consumption carried out by university women, but also to go one step further, and evaluate which variables (expectancies, motives, and consequences experienced by subjects after consuming alcohol) are that make it more likely to engage in BD.

When assessing the determinants of drinking–expectancies, motives and experienced consequences–comparing women who engage in BD with those who consume alcohol at a lower level, the first ones score higher on all determinants, as in previous literature (Iwamoto et al., 2018; McCaul et al., 2019). Specifically, among women who consume alcohol and engaged in BD, it is the positive expectancies that show greater relevance, compared to the negative ones (Watts et al., 2015; Young et al., 2015; Jacobs and Jacobs, 2016; Iwamoto et al., 2018; Kim, 2018). On the other hand, among the reasons for consumption, BD female university students allude to social motives, followed by coping with depression, and enhancement motives. The importance they give to social and enhancement motives for consumption supports the results of other studies in which most university students report that they drink for social and fun reasons (Patrick et al., 2021; Richards et al., 2021).

In the case of coping motives, most research carried out to date (Cooper et al., 2016; Hussman, 2018; Bacio, 2021; Richards et al., 2021) does not allow differentiating the type of emotion that is coped with through consumption (anxiety and/or depression) as it is assessed jointly. In this work, by using an assessment instrument that differentiates these two factors -those of anxiety and those of depression-, it has been possible to determine that it is the coping motives of depressive moods (for example, *to forget my worries; to stop thinking negatively about myself; to stop feeling pessimistic about the future*.) that contribute to explaining different risky consumption patterns. The relevance of anxiety-related coping motives (e.g., *to relax; because it makes me feel more self-confident; because it helps me when I am nervous*), which also show the lowest reliability coefficient in all samples tested, will have to be further explored in future research (Grant et al., 2007; Mezquita et al., 2011).

To achieve the second objective, logistic regression models allow us to know the strength of the association through the OR of the risk factors with the effect studied independently and to know the predictive value of each of them. As a descriptive method, it allows us to study the occurrence of a given event in a group of individuals. Once the model is fixed, the probability of performing BD can be estimated based on a series of variables, being very useful in prevention. This work therefore adds that possibility in the field of risky alcohol consumption.

From the set of all the variables that have been included in the model, only social and conformity motives, ACCE, and positive expectancies are useful in classification BD in approximately 90% of the cases. It is worth noting that the enhancement and coping with depression motives that had a relevant weight among female consumers, especially among those who engage in BD, are relegated to second place when they enter our regression analysis.

The results obtained confirm, as stated by other researchers (for example Corbin et al., 2008; Capron and Schmidt, 2012), the adequacy of including the consequences that have been experienced with alcohol consumption over the last year among the possible independent variables, given that they provide a unique variation when assessing the probability of engaging in BD, beyond the contribution of consumption expectancies and motives. In addition, it is the variable that shows the highest coefficient of all those that have been evaluated in this study.

On the other hand, these results reinforce the findings of previous research in which it has been proven that social motives for consumption are closely linked to frequency and amount of alcohol ingested (Weybright et al., 2016; Richards et al., 2021), as well as the BD pattern (Patrick et al., 2020; Valencia-Martín et al., 2020; Richards et al., 2021). Despite this, research has focused more on extracting predictive models of the influence of coping and enhancement motives, indicating that they are more stable over time and that they are associated with a greater presence of negative consequences. Motives of a more external nature such as social is left out (Cooper et al., 2016) despite being the most present among university students (Patrick et al., 2021; Richards et al., 2021). The present study reinforces the importance of contemplating social motives, given that the higher the score in this type of motives, the greater the probability of BD, unlike the more internal reasons (coping and enhancement) in which this relationship is not so evident.

Regarding the results on conformity motives, despite the inconsistency shown by this construct (Cooper et al., 2016), our results coincide with those of previous research in which a negative relationship with alcohol consumption (Grant B. F. et al., 2009; Richards et al., 2021) and especially with risky alcohol consumption has been shown (Cooper, 1994; Kuntsche et al., 2008; Bainter and Ackerman, 2021). It is striking that Hussman (2018) finds conformity motives relevant for female university students engaged in BD. However, this result can be explained by her multidimensional assessment of this construct, using different scales that address both traditional norms of femininity (desire to be thin, investment in appearance, maintenance of social relationships) and other internalized ideologies that are relevant in emerging adulthood (objectification of the body, inauthenticity of relationships) (Hussman, 2018; Iwamoto et al., 2018).

It is worth noting that the group of female university students who acknowledged having experienced more than 25 of the consequences included in the ACCE, those with the highest risk, presented a lower probability of engaging in BD than the group of consumers rated as moderate risk with this same instrument. However, this is easily understandable if the heterogeneity of consumers included under the BD category is taken into account. Limiting the BD to a number of grams consumed in a time interval implies that this group includes consumers of the minimum amount required, but also those who double or even triple this amount. Many of the female university students who double or triple this amount already meet the criteria for an alcohol use disorder, far exceeding occasional consumption in the form of BD. It should also be added that as the years of BD consumption increase, other lesser amounts of consumption are added, which make it easier for young people to progress within the addictive process. It would be convenient in future studies to include a registry of alcohol consumption that is not limited to BD, but that would allow for classifications to be more adjusted to the real consumption pattern those young women are carrying out, also contemplating possible weekly and even daily risk consumption in those cases that are more advanced in the addictive process.

The information extracted from this study allows us to identify the potential points to work on to reduce the probability of BD. Mainly, it highlights the adequacy of applying intervention measures focused on the consequences that are experienced when consuming alcohol, paying special attention to how these can deteriorate one’s daily life. In addition, the percentage explained over the BD obtained by motives and expectancies warns of the need to work on these consequences, relating them especially to social motives and to the positive expectancies that modulate decision-making when engaging in this pattern of consumption. It would be important to reflect on the extent to which the motives and expectancies that are linked to BD contradict the actually experienced consequences and whether these are permanent or not.

Furthermore, despite the low and negative weight of the conformity motives, it is important to increase the level of awareness that these young women have regarding the influence that social pressure may be having on deciding to engage in BD. It requires self-exploration and reflection on the false freedom they believe they have when they make the decision to engage in BD.

This section can be concluded by highlighting that with only four variables -consequences, positive expectancies, social motives, and conformity motives- it is possible to classify 26.3% of BD in university women, surpassing the results of previous investigations in which 9.5% (Motos-Sellés et al., 2015) and 18% of the variance of weekly risky consumption was explained (Ibáñez<suffix>I</suffix>, Moya et al., 2010), based on age of onset of alcohol consumption and personality variables.

## Limitations and Future Directions

The limitations of this study include having used self-report measures to evaluate the different variables, thus assuming possible biases in the responses issued, including social desirability (Kaya et al., 2016). This could be the reason why a small group of women evaluated, despite being classified within the high-risk group in the ACCE, have not registered a pattern of consumption adjusted to this reality.

Although the sample with which the study was carried out is large, it was obtained without random selection or stratified sampling, so our results can only be generalized to groups of university women within a certain age range. With stratified sampling, a sample with more balanced BD/non-BD group sizes could be achieved. In addition, it would be very useful to establish categories within the BD group, given that consuming the minimum to enter this category (60 grams) is not the same as consuming other amounts in this sample (for example, 130 grams). At the moment, regardless of consumption, a person is included in the BD group if the 60-gram barrier is exceeded.

One last aspect to consider would be improving the evaluation of one of the most controversial constructs to date, conformity motives. Following the proposal of Hussman (2018) and Iwamoto et al. (2018), using instruments for women samples that include female normative aspects that better explain their need for adjustment could be considered.

Moreover, to improve the understanding of this behavior, it’s recommended to carry out longitudinal studies in which the usefulness of this study for predictive purposes can be tested, as well as the inclusion of more variables that can account for the pattern of behavior.

## Data Availability Statement

The raw data supporting the conclusions of this article will be made available by the authors, without undue reservation.

## Ethics Statement

This study was undertaken in compliance with Spanish legislation (approved by the Department of Education) and the code of ethics for research involving human subjects outlined by the University of Valencia Human Research Ethics Committee. The patients/participants provided their written informed consent to participate in this study.

## Author Contributions

M-DS-B performed the analysis. M-TC-T, J-AG-C, and PM-S wrote the first draft of the manuscript. All authors contributed to the study conception and design, material preparation and data collection, commented on previous versions of the manuscript, read, and approved the final manuscript.

## Conflict of Interest

The authors declare that the research was conducted in the absence of any commercial or financial relationships that could be construed as a potential conflict of interest.

## Publisher’s Note

All claims expressed in this article are solely those of the authors and do not necessarily represent those of their affiliated organizations, or those of the publisher, the editors and the reviewers. Any product that may be evaluated in this article, or claim that may be made by its manufacturer, is not guaranteed or endorsed by the publisher.
